# Differential analysis of glioblastoma multiforme proteome by a 2D-DIGE approach

**DOI:** 10.1186/1477-5956-9-16

**Published:** 2011-04-06

**Authors:** Brigitte Collet, Nathalie Guitton, Stephan Saïkali, Tony Avril, Charles Pineau, Abderrahmane Hamlat, Jean Mosser, Véronique Quillien

**Affiliations:** 1 Centre Régional de Lutte contre le Cancer; Rennes, France; 2 High-Throughput Proteomics Platform Biogenouest; 263 avenue du Général Leclerc, Campus de Beaulieu, Rennes, F-35042, France; 3 CHU Pontchaillou; Rennes, France; 4 CNRS UMR6061 Institut de génétique et de développement Rennes, France

## Abstract

**Background:**

Genomics, transcriptomics and proteomics of glioblastoma multiforme (GBM) have recently emerged as possible tools to discover therapeutic targets and biomarkers for new therapies including immunotherapy. It is well known that macroscopically complete surgical excision, radiotherapy and chemotherapy have therapeutic limitations to improve survival in these patients. In this study, we used a differential proteomic-based technique (2D-Difference Gel Electrophoresis) coupled with matrix-assisted laser desorption/ionization-time of flight (MALDI-TOF) mass spectrometry to identify proteins that may serve as brain tumor antigens in new therapeutic assays. Five samples of patients presenting a GBM and five samples of microscopically normal brain tissues derived from brain epileptic surgery specimen were labeled and run in 2D-PAGE (Two-Dimensional Polyacrylamide Gel Electrophoresis) with an internal pool sample on each gel. Five gels were matched and compared with DIA (Difference In-gel Analysis) software. Differential spots were picked, in-gel digested and peptide mass fingerprints were obtained.

**Results:**

From 51 protein-spots significantly up-regulated in GBM samples, mass spectrometry (MS) identified twenty-two proteins. The differential expression of a selected protein set was first validated by western-blotting, then tested on large cohorts of GBM specimens and non-tumor tissues, using immunohistochemistry and real-time RT-PCR.

**Conclusions:**

Our results confirmed the importance of previously described proteins in glioma pathology and their potential usefulness as biological markers but also revealed some new interesting targets for future therapies.

## Introduction

Malignant gliomas are the most common human primary brain tumors, glioblastoma multiforme (GBM) being the most aggressive and lethal form. Median survival of patients affected by GBM is around 15 months and nearly 75% of them will die within 18 months following diagnosis [1] despite treatment combining complete excision then chemo- and radiotherapy, pointing to the need for non-conventional therapies. Research has thus focused on specific strategies targeting intracellular signaling pathways [2], specific surface molecules, antiangiogenesis therapy [3], immunotherapy [4] and combinatorial approaches. Many reports identifying genes [5] and proteins which can be used to distinguish between GBM and either other gliomas or non tumorous brain tissue have increased our knowledge of this pathology, but a combination of genomic, proteomic and transcriptomic data will be needed to identify new therapeutic targets or biomarkers.

Proteomic approaches were most widely based on methods using differential expression on 2D-PAGE gels [6] or, more recently, two dimensional chromatography [7] followed by mass spectrometry protein identification. The 2D-DIGE (two dimensional difference gel electrophoresis) technology, using a mixed-sample internal standard, is now recognized as an accurate method to determine and quantify human proteins, reducing inter-gel variability and simplifying gel analysis. In the present study, GBM (T) and non tumorous (Nt) brain samples were labeled and fractionated using 2D-DIGE. Mass fingerprints of differentially expressed proteins were acquired with a MALDI-TOF/TOF spectrometer. Western-blot experiments, or immunohistochemical analysis for the largest series, were applied to confirm the up-regulation of selected proteins. The relationship with mRNA expression of coding genes for some over expressed proteins was analyzed by quantitative RT-PCR.

## Results

### Detection and identification of differentially-expressed proteins in GBM versus non-tumorous brain tissue

**Figure 1:**
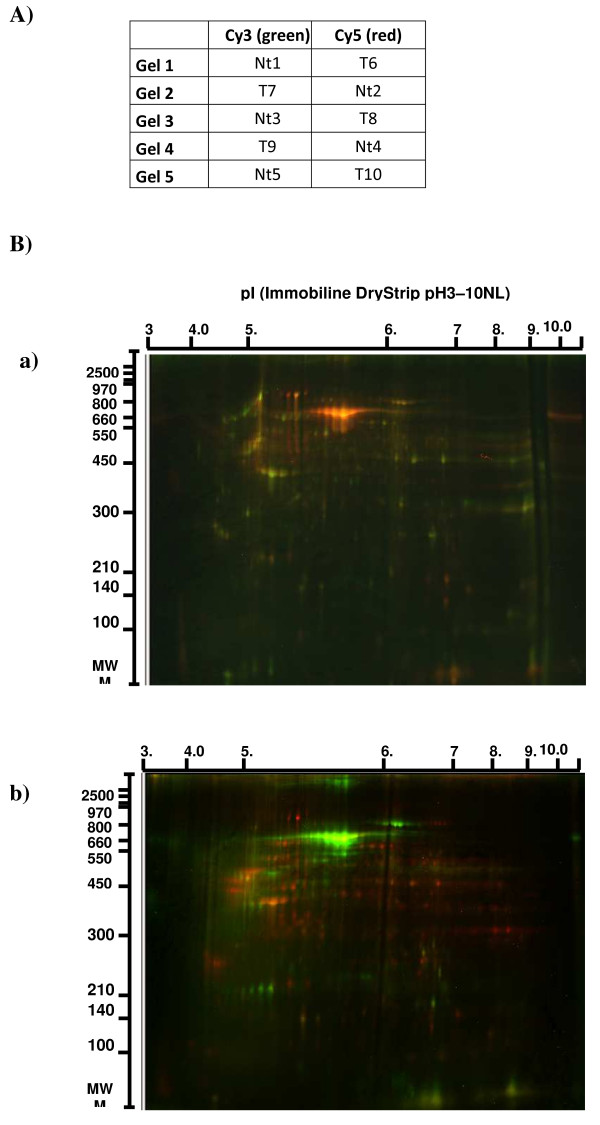
**DIGE analysis on a series of 5 GBM samples (T) and 5 non tumorous samples (Nt)**. A) Experimental design. B) Examples of two DIGE gels: **a) **Gel 1: the GBM sample (T6) was labeled with Cy5 Dye (red spots) and the control sample (Nt1) with Cy3 Dye (green spots). **b) **Gel 2: the GBM sample (T7) was labeled with Cy3 Dye (green spots) and control sample (Nt2) with Cy5 Dye (red spots). Merge spots appeared in yellow. In all gels, internal standard was labeled with Cy-2 Dye (not visible in these images).

2D-DIGE was used to identify proteins differentially expressed in GBM *versus *non tumorous samples. The two-group experimental design (Figure 1A) using a mixed internal standard enabled us to normalize spot volumes from each sample, to perform inter-gel comparisons and to recognize statistically significant inter-spot variations. A representative set of overlaid 2D-DIGE images gels is given in Figure 1B.

On each analytical 2D gel, an average of 700 spots corresponding to proteins with a pI between 3 and 10 and a molecular weight from 10 to 250 kDa was detected and 457 spots were matched across the 5 gels. A total of 51 spots showed statistically significant differences (Student's t-test p-value ≤ 0.05) in protein expression between the two populations with an average ratio > 2 and a statistical power of 0.88. These 51 spots were up-regulated in GBM. Only protein spots on silver or Coomassie Blue-stained gels manually matching with the corresponding DIGE images were processed. Thirty protein-spots were identified, corresponding to 22 different proteins (Table 1). This discrepancy is explained by the fact that some proteins, such as hemoglobin, DRP-2 (dihydropyrimidinase related protein 2, or collapsin response mediator protein 2, CRMP-2) or ER-60 (Endoplasmic reticulum resident protein 60 or protein disulfide-isomerase A3), are found in several spots while in other circumstances two proteins can contribute to the same spot. Results of DIGE analysis (average ratio glioblastoma/normal brain tissue (GBM/NT), T-test), Mascot search (matched peptides, % of coverage and Mascot score) and protein characteristics (accession number, theoretical Mt and pI) are listed for each protein in Table 1.

**Table 1:** Differentially expressed proteins in GBM samples identified by MALDI mass spectrometry after DIGE analysis*

Average ratio GBM/NT(a)	T-test (b)	Protein name	Species	Mt Th.( c)	PI Th. (d)	Accession No. (gi) (e)	Matched Peptides (f)	Coverage % (g)	Score(h)
7.33	0,0060	hemoglobin	*Homo Sapiens*	15834	6.76	61679606	11/82	80	100

6.52	0.0065	dihydropyrimidinase -related protein 2	*Homo Sapiens*	62711	5.95	4503377	24/73	55	225

6.49	0,0058	apolipoprotein A-1	*Homo Sapiens*	28061	5.27	90108666	17/69	50	153

6.47	0,035	human serum albumin	*Homo Sapiens*	67174	5.57	55669910	26/59	54	263

6.19	0,0078	alpha-2-globin	*Homo Sapiens*	15174	8.73	1335076	9/55	69	86

5.88	0.024	tubulin beta 2	*Homo Sapiens*	50274	4.78	4507729	22/73	43	207

5.83	0,0015	ubiquitin carboxyl-terminal hydrolase isozyme L1	*Homo Sapiens*	25151	5.33	21361091	10/40	59	131

5.83	0,0022	ER-60 protease	*Homo Sapiens*	57160	5.98	1208427	17/40	34	170

4.60	0,0085	MTHSP75 (GRP-75)	*Homo Sapiens*	74019	5.9	292059	26/72	49	208

4.29	0.020	dihydropyrimidinase-related protein 3	*Homo Sapiens*	62323	6.11	4503379	12/29	27	76

4.26	0,048	vimentin variant	*Homo Sapiens*	53708	5.06	62896523	25/100	59	172
		higly similar to glial fibrillary acid protein (GFAP)	*Homo Sapiens*	49533	5.84	34536332	26/100	63	169

4.14	0,040	ACTB protein	*Homo Sapiens*	40536	5.55	15277503	12/57	45	93

4.02	0.050	transthyretin variants	*Homo Sapiens*	13806	5.57	2098255	10/42	81	153

3.59	0,049	human manganese superoxide dismutase mutantQ143n (MnSOD)	*Homo Sapiens*	22290	6.86	2780818	10/59	46	95

3.57	0,034	glyceraldehyde-3 phosphate dehydrogenase	*Homo Sapiens*	24776	8.68	89573929	8/39	47	89

3.32	0,021	heat shock protein 27	*Homo Sapiens*	22427	7.83	662841	10/67	57	99

2.83	0,050	mitochondrial aldehyde dehydrogenase	*Homo Sapiens*	54394	5,6	6137677	17/71	43,3	134

2.76	0,037	tubulin alpha -1C	*Homo Sapiens*	50548	4.96	14389309	15/66	49	138

2.76	0,0084	tubulin alpha	*Homo Sapiens*	33321	5.86	37492	8/30	22	71

2.74	0,040	glutamate carboxypeptidase	*Homo Sapiens*	52700	5.7	15620780	14/40	38	143

2.57	0,024	LAP 3 protein (leucyl aminopeptidase)	*Homo Sapiens*	54724	6.80	37588925	15/46	38	149

* CyDye images were analyzed by BVA and spots that showed statistically significant differences (Student's *t*-test) in intensity between the control and the GBM groups are listed. (a) Average ratio GBM/NT: The average ratio value indicates the standardized volume ratio between the two groups: GBM and non tumorous patients. A two-fold increase is represented by 2. (b) T-test: Student's T-test *p *value: Only proteins with ratios showing significant differences (p£0.05) were retained. (c):Mt th: Theoritical relative mass. (d) PI th: Theoritical isoelectric point. (e) GeneBank sequence identification number(f): Number of matched peptides versus total number of peptides. (g): Percent coverage of the protein. (h) Mascot score: Protein scores greater than 81 are significant (p < 0.05).

GBM-overexpressed proteins included several blood proteins. Tumors spots for hemoglobin, apolipoprotein A1, serum albumin, and alpha-2 globulin were five times larger than the same spots from non-tumorous extracts.

Transthyretin was also four times more abundant in GBM. Two members of the dihydropyrimidinase family, DRP-2 and DRP-3 (or CRMP-4), were identified with an average GBM/NT ratio of 6.5 and 4.29 respectively. Three chaperone proteins HSP 27 (heat shock 27 kDa protein), GRP-75 (75 kDa glucose-related protein or heat-shock 70 kDa protein 9 also named mortalin), and ER 60 were also over-expressed in GBM.

Some of the presently identified proteins have previously been described as brain tumor markers [8], i.e. glyceraldehyde-3-phosphate dehydrogenase (GAPDH), hemoglobin, tubulin beta, HSP 27, manganese superoxide dismutase Mn-SOD, vimentin, albumin, apolipoprotein A-1, ubiquitine carboxyl-terminal hydrolase L1 (UCH-L1) and glial fibrillary acid protein (GFAP), or implicated in neurodegenerative diseases, i.e. UCH-L1 in both Huntington's and Parkinson's diseases, apolipoprotein A-1 and DRP-2 in Alzheimer's disease [8].

### Validation of 2D-DIGE results by western blot analysis

**Figure 2:**
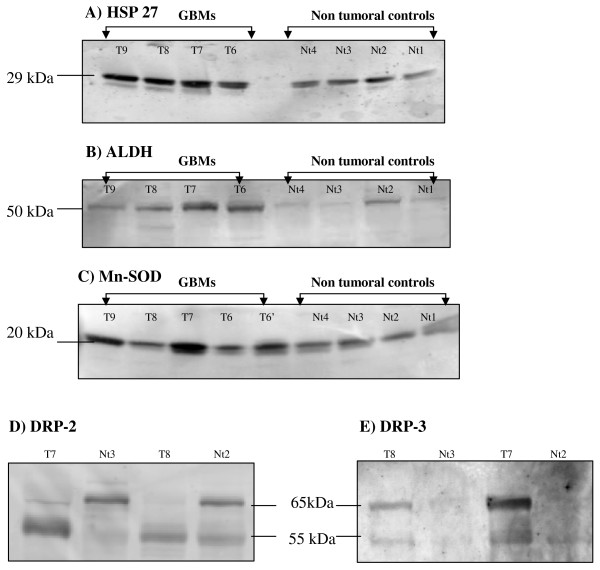
**Selected proteins expression analyzed by western-blot**. GBM (T) and non tumoral control (Nt) protein extracts were loaded on 1D SDS-PAGE gels together with biotinylated molecular weight markers and transferred on Hybond-P membranes, then stained with primary and secondary-HRP antibodies or Streptavidin-HRP. Immunoreaction was revealed using ECL. Protein expression was tested for HSP 27 (A), ALDH (B), Mn-SOD (C), DRP-2 (D) and DRP-3 (E).

To validate the results of the DIGE analysis, five proteins, selected on the basis of interesting biological functions and high-fold changes, were tested in western blotting experiments with specific antibodies available commercially, on tumor protein extracts from GBM and control patients already used in 2D-DIGE. Equal amount of proteins from each sample were loaded. As shown in Figure 2, the expression levels for HSP 27 (29 kDa), ALDH (aldehyde dehydrogenase, 50 kDa), and Mn-SOD (20 kDa) were higher in GBM (n = 4 for Hsp27 and ALDH and n = 6 for MnSOD) in comparison to their expression in non-tumorous samples, thus confirming the DIGE analysis.

Two bands (55 kDa and 65 kDa) were found for DRP-2 and DRP-3. DRP-2 protein was expressed with a strong signal at 55 kDa in GBM (n = 2) and a slight signal at 65 kDa while the contrary was seen for non-tumorous samples. DRP-3 protein expression was also different in GBM (n = 2) with a high signal at 65 kDa and a slighter one at 55 kDa and non-tumorous samples where no or faint bands could be found.

### *In situ *validation of proteomic data by immunohistochemistry

**Figure 3:**
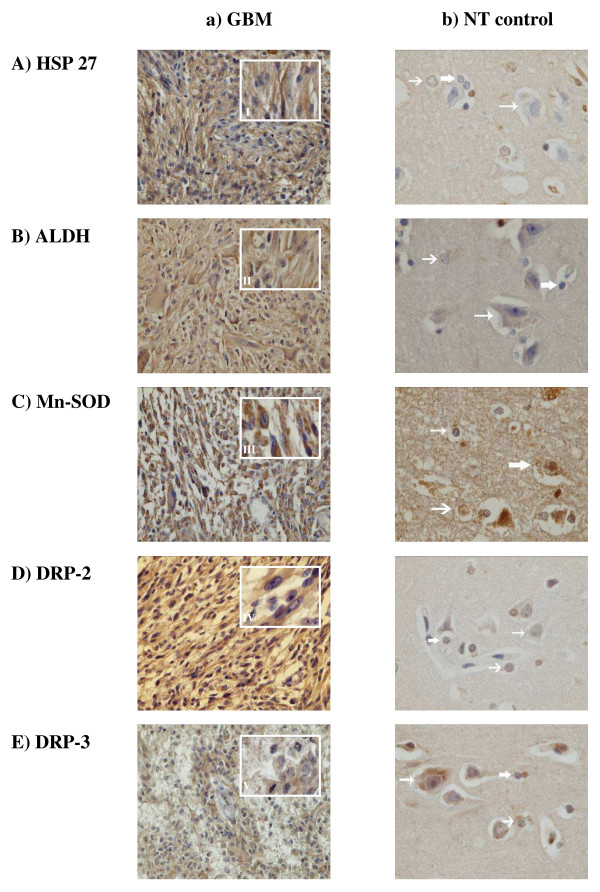
**Selected proteins expression analyzed by immunohistochemistry**. GBM (a) and non tumor control (b) paraffin-embedded sections were stained with anti-HSP 27 (A), -ALDH (B), -Mn-SOD (C), -DRP-2 (D) and -DRP-3 (E) antibodies. Normal brain staining and one representative sample are shown for each antigen. In control samples, the major cell types are indicated with arrows: the thickest arrows designate oligodendrocytes, the intermediate arrows astrocytes and the narrow ones neurons. Magnifications for GBM samples were x 400 with a window (I, II, III, IV,V) showing magnified small region (x 1000). Control samples were magnified x 1000.

**Figure 4:**
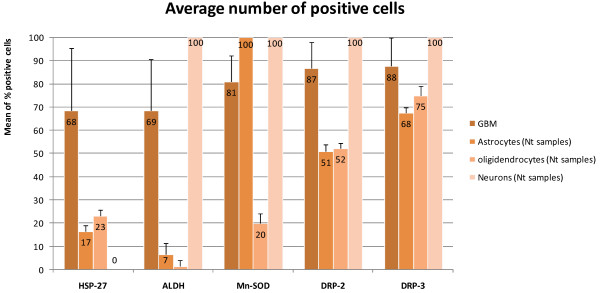
**In situ validation of the protein expression data obtained by immunohistochemistry**. For each antibody, 1000 tumor cells were counted and results were expressed as a percentage of positive cytoplasmic staining in two different and most expressive areas. Bars represent standard deviation of results obtained with the 25 GBM and the three populations (astrocytes and oligodendrocytes and neurons) of 3 non-tumorous samples (Nt samples). The expression of four proteins tested (HSP 27, ALDH, DRP-2, DRP-3) was higher in GBM cells than in normal astrocyte or oligodendrocyte populations. All neurons were positive (100%) except for HSP 27 (0%).

Immunohistochemical studies were carried out with the five selected proteins already tested in western blot analysis, namely HSP 27, ALDH, Mn-SOD, DRP-2 and DRP-3, revealed in 25 GBM and three non-tumoral brain samples. The results confirmed their high expression in GBM. Figure 3 shows a representative paraffin-embedded section and the respective non-tumorous control for each protein. Immunohistochemical studies gave the distribution of these proteins by cell category of the normal parenchyma: astrocytes, oligodendrocytes, neurons. All neurons (100%) were positive for ALDH, Mn-SOD, DRP-2 and DRP-3 (Figure 4) except HSP 27 (0%). DRP-family molecules were strongly expressed in normal astrocytes and oligodendrocytes (51% and 52% positive cells respectively for DRP-2 and 68% and 75% for DRP-3) however this expression was higher in GBM cells (87% for DRP-2 and 88% for DRP-3). HSP 27 and ALDH were also more expressed in GBM (68% and 69% respectively) than in astrocytes (17% and 7%) or oligodendrocytes (23% and 2%).

### mRNA expression analysis of genes corresponding to the up-regulated proteins in GBM samples

Real-time RT-PCR assays on a series of 50 GBMs and nine non-tumorous samples was performed to examine the levels of transcript for HSP 27 (*HSPB1*), MnSOD (*SOD2*), ALDH (*ALDH2*), DRP-2 (*DPYSL2*), DRP-3 (*DPYSL3*) plus GRP-75(*HSPA9*) and UCHL-1(*UCHL1*).

mRNA of frozen tissues was isolated and the amplification of each selected genes, compared with GAPDH amplification, was normalized via a pool of previously used non-tumorous brain samples (or "control") [9]. mRNA expression for GAPDH was identical for control and GBM and none of the nine non-tumorous samples tested against this control was considered as positive in our study (data not shown).

**Figure 5:**
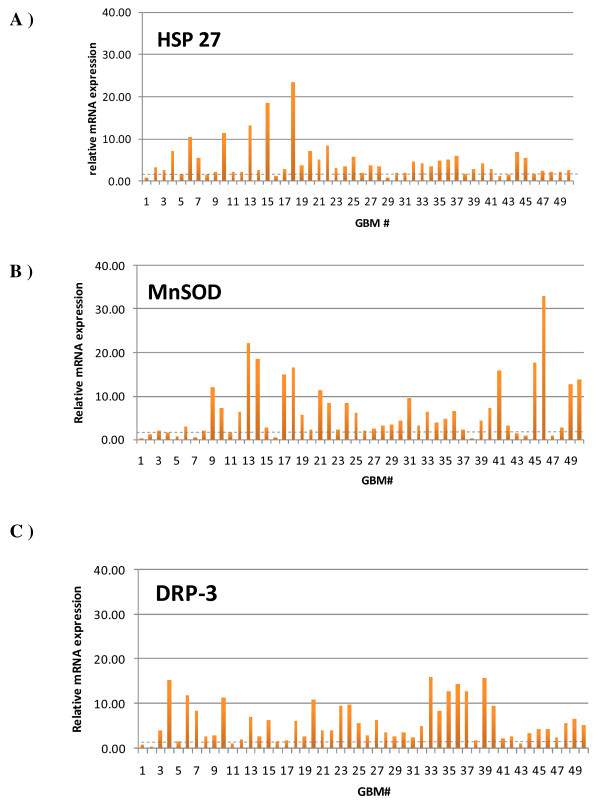
**mRNA expression in 50 GBM samples analyzed by real-time RT-PCR**. Results are expressed as the relative HSP 27 (A), Mn-SOD (B) and DRP-3 (C) mRNA expression compared with the same pool of non-tumorous samples.

UCH-L1 expression was not increased in the GBM samples. ALDH, GRP-75 and DRP-2 disclosed mild positivity with respectively 4%, 4% and 6% of the GBM samples exhibiting an increase in mRNA expression more than 2-fold greater than the reference. In contrast, HSP 27, MnSOD and DRP-3 were clearly up-regulated with respectively 32%, 44% and 46% of the GBM samples having a 5-fold increase in mRNA expression compared with the pool of non-tumorous brain samples (Figure 5).

## Discussion

Extensive proteomic studies of human GBM have emerged in the last decade allowing comparison between GBM and normal brain proteomes and identification of differences in protein expression and profiles of diverse grades of gliomas (for review see [6,10]).

The aim of the present proteomic work was to highlight potential new glioblastoma tumor antigens thus extending the number of proteins already used and investigated, particularly in immunotherapy assays [9]. The 2D-DIGE technique was used to compare five GBM and five non-tumorous brain samples. Differential protein expression was analyzed by mass spectrometry. 2D-DIGE is a very powerful technique allowing controls and experimental samples to be run on the same gel together with an internal reference, each sample being labeled with a different cyanine. Using the DeCyder software, gel images can be analyzed to identify statistically significant differences in protein expression between different samples on a limited number of gels [11]. This technique is very useful for applications requiring accurate quantization and direct differential proteomic analysis of normal and abnormal tissues [12].

Twenty-two proteins were statistically highly expressed in GBM comparatively to non-tumorous brain samples. Some of them are involved in key metabolic pathways and suspected to participate in tumorigenic processes.

### Blood and cytoskeletal proteins: two major groups up-regulated in GBM

Blood proteins emerged as a significant group highlighting the high angiogenesis capacity of GBM. GBM are highly vascular tumours and this study analyzed the proteome of the whole GBM sample extract, without removing blood vessels. In a recent review [13], up-regulation of serum albumin and apolipoprotein-A1 were commented as the consequence of the blood brain barrier degradation in malignant gliomas. These results reinforce previous reports describing hemoglobin [8], apolipoprotein A-1 or albumin [8,14] as brain tumor markers up-regulated in GBM.

Transthyretin (TTR), a plasma protein described as thyroxin and retinol transporter, has recently gained interest in neurobiology and seems to represent a key protein in nervous system physiology [15]. Although TTR is synthesized in the choroid plexus, there are several unclear points concerning its other precise localizations and functions. In cerebrospinal fluid, TTR levels are raised and lowered in Parkinson's and Alzheimer's diseases respectively. Furthermore a physiological variant, transthyretin Thr119met chain, is highly expressed in malignant areas of GBM [16]. The biological significance of this GBM-TTR overexpression and either its implication in gliomagenesis or its possible therapeutic role need further investigations.

Another significant class of differentially expressed proteins is represented by cytoskeletal proteins: vimentin, alpha (6,3) and beta (2) tubulin, beta actin and GFAP are three- to six-fold increased in GBM. Beta-tubulins and microtubule components are encoded by a multigene family whose expression patterns are complex. In tumor tissues, most isotypes exhibited an altered expression which might represent markers of sensitivity for drug response [17]. GFAP, a well-known specific astrocytic marker, has been suggested as a serum diagnostic marker for GBM [18]. It has been also disclosed in different forms in high-grade gliomas (grade III and IV) but its overexpression has been linked with the lower grade [19]. In the present work, GFAP was identified in three spots, all of them being up-regulated in GBM.

### Mitochondrial and endoplasmic reticulum proteins: enzymatic and chaperone proteins involved in key pathways and dysregulated in GBM

In eukaryotic cells, mitochondria and endoplasmic reticulum form two endomembrane networks which interact closely with each other to control metabolic flow, protein transport, intracellular signaling and cell death. In normal glial cells, besides their essential role of adenosine triphosphate (ATP) generation to produce energy, mitochondria are also involved in the regulation of cellular proliferation and apoptosis. The important consequences of mitochondrial dysfunction in glioma in these three areas was highlighted in a recent study [20]. It is not surprising to find some mitochondrial or endoplasmic reticulum proteins differentially expressed in GBM and in non-tumorous samples. In the present work, DIGE analysis and immunohistochemistry study revealed that two mitochondrial enzymes, ALDH and Mn-SOD, are highly expressed in GBM: on average in a series of 50 GBM, 69% tumor cells were ALDH positive and 81% Mn-SOD positive. RT-PCR analysis also revealed a high level of transcripts encoding for MnSOD. Mn-SOD expression has recently been suggested as a potential GBM prognostic marker [21] because the lack of overexpression is found in three years survival group patients compared to short-term survival patients. These results confirmed earlier work which correlated shortest median survival with high Mn-SOD enzyme expression level in GBM patients [22].

The second mitochondrial enzyme, ALDH, has not, to our knowledge, been reported as a brain tumor marker, but ALDH activity, detected commonly with ALDEFLUOR assay, has been used for identification and isolation of adult stem cells and particularly neural stem cells [23]. Cancer stem cells - or tumor initiating cells (TICs)-, might be also identified in this way. Recently, ALDH expression, characterized by immunohistochemical staining of common epithelial cancers and their corresponding normal tissues, has been correlated with ALDH enzymatic activity. In tumors for which corresponding normal tissues expressed relatively low ALDH levels (breast, lung, ovarian or colonic cancer), ALDH can be proposed as a cancer stem cell marker [24]. Moreover, in breast and pancreatic cancers [25-27] the presence of positive cells has been related with poor survival, which was possibly explained by a greatest resistance to chemotherapeutic drugs as demonstrated for ovarian cancer cells [24]. In GBM, there is now much evidence that such tumor stem cells exist and despite several markers proposed for their identification (CD133, SSEA-1 [28], A2B5 [29]), no consensus has been reached. ALDH could therefore be a good candidate for best TIC enrichment in GBM.

GRP-75 (mortalin, MTHSP75), the mitochondrial chaperone Hsp70 kDa protein isoform 9, a major mitochondrial protein, plays a central role in protein import and export. It could also localize in endoplasmic reticulum and contribute to modulation of the stress response. GRP-75 was previously linked with malignant progression of low-grade astrocytoma [30] and an increased expression of these chaperones contribute to tumorigenesis [31]. Our 2D-DIGE analysis confirmed a higher expression in GBM samples for GRP-75 and also two additional chaperone proteins: the reticulum endoplasmic protein disulfite isomerase (ER-60, ERp57 or GRP-57) and the heat-shock protein HSP 27. In brain, HSP 27 is linked with high-grade gliomas [32] or high malignancy human glioma cell line U87MGΔEGFR [33]. In cancer cells, HSP 27 is currently considered to participate in oncogenesis and in resistance to chemotherapy [34]. Conversely, ER-60 was previously reported as less expressed in GBM relatively to low-grade astrocytomas [32,35], although its key role in the control of newly synthesized glycoproteins during oncogenic transformation has been recently assessed [36]. Additionally, ER-60 is involved in the modulation of STAT-3 signaling and could contribute to a neoplastic diseased state. These findings highlight the importance of mitochondrial and reticulum endoplasmic proteins as targets for new therapies and suggest a particular study of these organelles via specific proteome analysis. In a recent paper, [13] a review of technical limitations in actual glioma proteomics shows the interest of sub-proteomes and particularly mitochondrial proteomes.

### Dihydropyrimidinase proteins: importance of isoforms

Two dihydropyrimidinase proteins overexpressed in GBM were selected and retained for validation experiments by western blot, immunochemistry and RT-PCR analysis. DRP-2 (CRMP-2/Ulip2) and DRP-3 (CRMP-4/Ulip 1) are members of collapsing response mediator proteins (CRMP) involved in regulation of neurite guidance and synapse formation; they are highly expressed during brain development and rarely in adult brains [37] except DRP-2 which can be detected until adulthood in several types of specialized neurons in the hippocampus, cerebellum and dorsal root ganglion. CRMP family members (DRP-1 to -5) have been essentially studied in the context of neurodegenerative diseases [38-40] where their expression is often altered. Nonetheless, some reports respectively outlined DRP-2 [8] down-expression and DRP-3 [14] up-regulation in GBM. In agreement with the later, DRP-3 which was identified in 2D-gels in only a single spot (65 kDa and 6.3 for MW and pI respectively) was confirmed up-regulated by western blot (65 kDa band), immunohistochemistry and RQ-PCR analysis. It was not so obvious for DRP-2: DIGE-study of GBM individualized four overexpressed spots: two spots with an apparent mass of 65 kD and pI around 5.9 and two acidic 55 kDa forms with 5.3 pI. In western blots studies, a 65 kDa-DRP-2 was over expressed in normal brain while in GBM it represented a lighter isoform (55-60 kDa). RQ-PCR results revealed that protein overexpression was not linked to a coding gene up-regulation. In addition, immunochemistry, which could not differentiate DRP-2 isoforms, confirmed that 87% of GBM cells were positive compared to 51% of astrocytes and 52% of oligodendrocytes in normal brain. The DRP family is known to be highly phosphorylated [41,42]. Depending on study design and experimental techniques, the molecular weight of phosphorylated or non- phosphorylated forms of DRP-2 has been reported to vary from 55 kDa to 70 kDa [43,44]. Other post-translational modifications such as myristilation, methylation or esterification can alter the pI of the proteins, and glycosylation and prenylation can alter their molecular weight [8]. In addition, some light forms (55 kDa) could be breakdown products [45]. Our findings revealed that DRP-2 was overexpressed in GBM, and that two isoforms could discriminate GBM from non-tumorous brain tissue. Indeed, additional work is necessary to analyze and determinate the nature of translational modifications responsible for these isoforms and confirm the 55 KDa form as a potential glioma marker.

In our study, we present five proteins that are also regulated in neurodegenerative diseases: UCHL-1, transhtyretin, Apolipoprotein A1, DRP-2 and DRP-3. Altered expression of these proteins in Alzheimer's disease (AD), Parkinson's disease (PD) or Down Syndrome (DS) may reflect their importance in brain physiology or may be the result of stress generated by the disease. UCHL-1 is down-regulated in AD and in PD [46], transthyretin is raised in PD, but lowered in AD. High levels of Apolipoprotein A1 were found to be associated with neurodegeneration in AD. Concerning DRP-family, DRP-2 was increased in paired helical filaments and was related to the loss of neurofibrillary tangles-free neurons in AD ([40]. In another work [38], DRP-2 was found down-regulated at the mRNA level in DS and AD. In contrast, at the proteomic level, an increased ratio of 55 kDa to the 65 kDa forms in AD and DS brains suggested decreased phosphorylation of the DRP-2 protein. Differential expression in spots for DRP-2 and DRP-3 was reported in the study of R. Weitzdoerfer *et al *[39] in a fetal DS brain analysis: increased levels of one spot assigned to DRP-3 and decreased levels of spots assigned to DRP-2. More studies about these proteins and their isoforms would be necessary to conclude about their use as markers in GBM.

Overall, this work underlines the up-regulation of several GBM-related proteins; this was confirmed by western blot and immunohistochemistry techniques in some instances. These proteins might be attractive clinical biomarkers, linked or not with survival, or a specific GBM immunotherapy target. It could be also interesting to explore specifically the mitochondrial or endoplasmic reticulum GBM proteome which might open the way to novel fields of therapeutic advances.

## Materials and methods

### Samples

GBM samples were obtained from patients admitted in the Neurosurgery Department of Rennes University Hospital and collected in accordance with the French regulations. For the entire study, 66 patients were included, with newly-diagnosed, untreated, primary glioblastoma (7 being classified as giant cell glioblastoma,) and two patients with a secondary glioblastoma. Twenty-three patients received only radiotherapy after surgical resection, when 27 patients received radiotherapy with concomitant temozolomide followed by adjuvant temozolomide (the current standard treatment). Other patients were treated after surgical resection with radiotherapy followed by different chemotherapeutic regimens.

2D-DIGE and western blot analysis were conducted on samples arising from five men (age range 43 - 72 years, mean: 60 years), classified as primary glioblastoma. The validation cohort for immunochemistry and RT-PCR analysis was composed of 34 men and 30 women, (age range 35 - 75 years mean: 59 years). Some patients were tested with both RT-PCR and immunochemistry. Non-tumorous brain tissues were obtained from normal areas (either grey or white matter) of brain tissues removed from patients undergoing non-tumor epileptic surgery. All samples were either conserved at -80°C for 2D-DIGE, real time RT-PCR and western blotting analysis, or formalin-fixed and paraffin-embedded for immunohistochemistry.

### Protein extraction

Cell lysates were prepared from five GBM and five non tumorous brain samples by mechanical disruption in 2.5 volumes of ice-cold lysis buffer (Tris 20 mM, pH7.5, CHAPS 4%, urea 8M (Sigma-Aldrich, St Louis, USA) and antiproteases cocktail (Complete EDTA-free tablets, Roche Diagnostics, Mannheim, Germany)). Samples were sonicated (6 cycles of ten seconds with relapse of 30 seconds in ice-bath) and centrifuged (15 000 g, 30 minutes, 4°C). Supernatants were ultra centrifuged at 108,000 g for 60 minutes at 4°C. Protein concentration, in the resulting supernatants containing cytosolic protein extracts, was determined using the Bradford protein assay. Aliquots (100 μg) were conserved at -80°C.

### Protein labeling with cyanin dyes

Cytosolic extracts were labeled with CyDyes DIGE Fluors developed for fluorescence 2-D DIGE technology (GE Healthcare, Bucks, UK) according to the manufacturer's recommended protocol. Briefly, 50 μg of each sample were minimally labeled with 400 pmol of amine-reactive cyanine dyes, Cy3 or Cy5, on ice for 30 minutes, in the dark. GBM and non-tumorous samples were labeled with Cy5 or Cy3 in a random manner to avoid dye-specific protein labeling, as described in Figure 1A. An internal pool, labeled with Cy2 fluorescent dye, was generated by combining equal amounts of all cell cytosolic extracts and was included in all the gels run in this study. The labeling reaction was quenched by incubation, for 10 minutes with 1 μL of 10 mM lysine (Sigma-Aldrich, ST Louis, USA) on ice, in a darkroom.

Following the labeling reaction, GBM extracts and their random non-tumorous tissue counterparts were combined together with the internal pool, and Destreak™ IEF buffer (GE Healthcare) was added to make up the volume to 450 μl prior to IEF (isoelectric focalisation) on five 24 cm gel strips.

### Two-dimensional SDS-PAGE

A first focusing isolectric electrophoresis was carried out on IPGphor™ system (GE Healthcare). Pre-cast immobilized pH gradient strips (pH 3-10 NL, 24 cm) were used for this first-dimensional separation for a total focusing time of 60 kV-h. After IEF, the IPG strips were incubated two times under ambient temperature, for 15 minutes in an equilibration solution (0.05M Tris-HCl pH 8.8, 6M Urea, 30% glycerol, 2% SDS and bromophenol blue) containing 65 mM DTT and 250 mM iodoacetamide respectively. Strips were directly applied on top of pre-cast 12% SDS-PAGE gels (GE Healthcare) and run in a vertical Ettan DaltSix system (GE Healthcare) for approximately 5 hours. Five gels were processed simultaneously.

### Gel imaging and data analysis

After SDS-PAGE, cyanine-labeled proteins were directly visualized using a Typhoon™ 9400 imager scan (GE Healthcare) in fluorescence mode. Cy2 images were scanned using a 488 nm laser and an emission filter of 520 nm. Cy3 images were scanned using a 532 nm laser and an emission filter of 580 nm. Cy5 images were scanned using a 633 nm laser and an emission filter of 670 nm. Each gel was scanned at 200 μm (pixel size) resolution and was processed using the DeCyder software V5.01 (GE Healthcare) allowing quantification, gel matching and statistical analyses.

The Differential In-gel Analysis module (DIA) was used for pair-wise comparison of the two samples (GBM and non-tumorous sample) on each gel, to exclude artifacts from gel images and differentially quantify the protein spots in the image. The Biological Variation Analysis module (BVA) was used to match the entire set of protein-spot maps from comparable gels simultaneously. Student's test (p < 0.05) was used for statistical analyses. Only spots with at least 2-fold changes in volume after normalization samples were defined as down- or up- regulated. The statistical power of the analysis was calculated similarly to Karp N. et al [47] and Engelen K. et al [48]. The standard deviation of the log_10 _(standardized abundance) per condition was calculated for each spots that have been matched across the 5 gels of the analysis. The median of these standard deviations was calculated in each condition to estimate the global variance of the replicates. The statistical power was then calculated for each condition using a tool available online (http://udel.edu/~mcdonald/statttest.html[49]), for a 2-fold change (effect size = 0.301), alpha = 0.05 and 5 replicates per group. After 2D-DIGE imaging and analysis, gels were post-stained with silver-stain [50] or Coomassie-blue. Gels were scanned (Image Scanner TM GE Healthcare) and stored in 1% acetic acid at 4°C until spot excision. Matching between silver- or Coomassie-blue stained gels and fluorescence maps was performed manually and pick lists were generated using the Image Master™ 2D Elite software (GE Healthcare).

### Protein identification by mass spectrometry

Silver-stained or Coomassie Blue-stained protein spots were excised from 2-D gels and processed using an Ettan™ Spot Handling Workstation (GE Healthcare). Gel plugs were washed 3 times in MilliQ water, once in 50% methanol/50 mM ammonium bicarbonate and once in 75% ACN to ensure complete removal of dye and detergent. After drying, gel pieces were re-hydrated for 60 minutes with 8.3 μg/ml (Silver-stained gels) or 16.6 μg/ml (Coomassie-blue stained gels) sequencing grade modified porcine trypsin (Promega, Charbonnières-les-bains, France) in 20 mM NH4HCO3. Extraction was performed in two successive steps by addition of 50% ACN and 0.1% TFA. Digests were dried out and dissolved in 2 mg/mL α-cyano-4-hydroxycinnamic acid in 70% ACN/0.1% TFA, before spotting onto MALDI targets (384 Scout MTP 600 μm AnchorChip™; Bruker Daltonics, GmbH, Bremen, Germany).

Peptide Mass fingerprints were acquired using a MALDI-ToF/ToF mass spectrometer (Ultraflex™; Bruker Daltonics, GmbH) and processed using the FlexAnalysis™ software (version 2.2; Bruker Daltonics, GmbH) for peak list generation and a first internal calibration with trypsin autodigestion peptides. Peak lists were then transferred to ProteinScape™ software (version 1.3; Bruker Daltonics, GmbH) for another automatic calibration based on a calibration list (related to the sample type and treatment) containing autolysis peaks and contaminants (keratins, polymers and background peaks). After re-calibration, an automatic trypsin and contaminants filtering and removal was performed in order to submit only m/z related to the sample and to obtain higher identification rates (ScoreBooster). Only the monoisotopic masses of tryptic peptides were then used to query NCBInr sequence databases (May 2008, 6493741 sequences) using the Mascot search algorithm (Mascot server version 2.1.04; http://www.matrixscience.com). Search conditions were as follows: initial rather open mass window of 70 ppm for an internal calibration, one missed cleavage allowed, modification of cysteines by iodoacetamide and methionine oxidation as variable modifications. Results were scored using the probability-based Mowse score (the protein score is -10 × log (P) were P is the probability that the observed match is a random event. In our conditions, a score greater than 81 indicated a significant identification (p < 0.05).

### Immunochemical validation of overexpression of selected proteins by western blotting

Cytosolic protein extracts (10-20 μg) were loaded on 12% polyacrylamide gels for 1D-SDS-PAGE together with biotinylated ECL western blotting molecular weight markers (Amersham-GE-Healthcare) and then electro-transferred on PVDF Hybond-P membrane (Amersham Biosciences). Equal amount of proteins (quantified by Bradford protein assay) for GBM and non tumorous samples were loaded on each gel and the good quality of transfer was visually verified with Red Ponceau staining of the membrane [51]. Non-specific sites were blocked in Tris-buffered saline (TBS) containing 5% (w/v) non-fat dry milk and blots were incubated with diluted primary antibodies in 0.1% Tween 20, 1% non-fat dry milk TBS. After washing in TBS, blots were incubated with peroxidase-conjugated secondary antibodies (Santa Cruz) diluted to 1/5000° in 1% non-fat dry milk TBS or Streptavidin-HRP for biotinylated markers. Immunoreaction was revealed using the enhanced chemiluminescence system ECL+ (GE Healthcare). Dilutions of specific primary antibodies were 1/200° for goat polyclonal anti-human HSP 27 (C-20), DRP-2/CRMP-2 (D-17), DRP-3/CRMP-4 (V-17). Concentrations of polyclonal anti-human ALDH1 (goat, Calbiochem) and Mn-SOD (rabbit, DD17, Sigma) were 2 μg/ml and 1.8 μg/ml respectively.

### Immunohistochemical procedure

Immunohistochemistry was performed on formalin-fixed and paraffin-embedded tissues, using 5-μm sections mounted on silanized slides. Antigen retrieval was performed using citrate buffer (pH 6.0) at 80°C Bain-marie (40 min) for DRP-3. The sections were incubated respectively at 20° for 30 and 60 minutes with diluted primary antibodies against HSP 27 and DRP-2 and overnight at 4°C with antibodies against DRP-3, ALDH, Mn-SOD (dilutions of 1:200, 1:25, 1:50, 1:200 and 1:800 for respectively HSP 27, DRP-2, DRP-3, ALDH, and Mn-SOD in antibody diluent of the Dako Cytomation Kit (Trappes, France)). A second incubation (60 minutes) with an anti-goat or anti-rabbit antibody (dilution 1:200, DAKO Cytomation) was followed by a peroxidase-staining procedure using the RTU Vectastain Elite ABC kit (Vector). Sections were counterstained with hematoxylin-eosin-safran. Microscopic analysis was performed using a Leitz-Diaplan microscope (Nuremberg, Germany). Negative controls were obtained in the absence of primary antibody. External positive controls were used for each staining: normal breast for HSP 27; dendate gyrus for DRP-2 and DRP-3; spleen for ALDH and myocard for Mn-SOD. A total of twenty- five GBM and three non-tumorous brain sampling were analyzed. For each antibody, 1000 tumor cells were counted and results were expressed as the percentage of cells with positive cytoplasmic staining in two different and most expressive analyzed areas.

### Quantification of gene expression by real-time RT-PCR

Total RNA was extracted from frozen tissues samples: 50 GBM and 9 non-tumorous brain as previously described [9]. In brief, sections of tumor samples, free of necrotic areas, were processed with the Rneasy Plus mini kit (Qiagen, Courtaboeuf, France). cDNA was prepared from 0.5 μg purified RNA (High Capacity cDNA Reverse transcription kit, Applied Biosystems, Courtaboeuf, France). Real-time-PCR was performed with a spectrofluorometric thermal cycler (ABI prism 7900, Applied Biosystems), following the manufacturer's recommendations. Primers and probes for HSP 27 (*HSPB1*), DRP-2 (*DPYSL2*), DRP-3 (*DPYSL3*), ALDH (*ALDH2*), MnSOD (*SOD2*),UCHL-1 (*UCHL1*) and GRP-75 (*HSPA9*) genes were purchased from Applied Biosystems (Assays-on-demand). Each point of data was run in duplicate. To normalize the data, GAPDH was chosen as an endogenous control. The comparative Ct method was used to determine relative gene copy numbers using the formula 2-ΔΔCt with a pool of non-neoplastic brain samples as reference.

## Abbreviations

AD: Alzheimer's disease; ALDH: Aldehyde dehydrogenase; 2D-DIGE: Two dimensional-difference gel electrophoresis; DRP-2: Dihydropyrimidinase related protein 2; DRP-3: Dihydropyrimidinase related protein 3; DS: Down Syndrome; ER-60: Endoplasmic reticulum resident protein 60; GAPDH: Glyceraldehyde-3-phosphate dehydrogenase; GBM: Glioblastoma multiforme; GFAP: Glial fibrillary acid protein; GRP-75: 75 kDa Glucose related protein; HSP 27: Heat shock 27 kDa protein; Mn-SOD: Manganese superoxyde dismutase; NT: Non tumorous brain tissue; PD: Parkinson's disease; UCHL-1: Ubiquitin carboxyl-terminal hydrolase L-1.

## Competing interests

The authors declare that they have no competing interests.

## Authors' contributions

BC carried out the proteomic, western blotting and transcriptomic studies and wrote the manuscript. SS carried out immuno-histochemical studies. NG and CP have made substantial contributions to the data acquisition and interpretation in proteomics and western blotting. AH has contributed to the obtaining of the samples. TA, VQ and CP participate in the design of the study and have been involved in writing the manuscript. JM contributed to the design of the study and to the transcriptomic study. All the authors have read and approved the final manuscript.

## References

[B1] Stark AM Nabavi A Mehdorn HM Blomer U Glioblastoma multiforme-report of 267 cases treated at a single institution Surg Neurol 2005 63 162 169 10.1016/j.surneu.2004.01.028 15680662

[B2] Sathornsumetee S Rich JN Designer therapies for glioblastoma multiforme Ann N Y Acad Sci 2008 1142 108 132 10.1196/annals.1444.009 18990124

[B3] Chi AS Norden AD Wen PY Antiangiogenic strategies for treatment of malignant gliomas Neurotherapeutics 2009 6 513 526 10.1016/j.nurt.2009.04.010 19560741 PMC5084187

[B4] Yamanaka R Dendritic-cell- and peptide-based vaccination strategies for glioma Neurosurg Rev 2009 32 265 273 10.1007/s10143-009-0189-1 19214609

[B5] Parsons DW Jones S Zhang X Lin JC Leary RJ Angenendt P An integrated genomic analysis of human glioblastoma multiforme Science 2008 321 1807 1812 10.1126/science.1164382 18772396 PMC2820389

[B6] Whittle IR Short DM Deighton RF Kerr LE Smith C McCulloch J Proteomic analysis of gliomas Br J Neurosurg 2007 21 576 582 10.1080/02688690701721691 18071984

[B7] Melchior K Tholey A Heisel S Keller A Lenhof HP Meese E Proteomic study of human glioblastoma multiforme tissue employing complementary two-dimensional liquid chromatography- and mass spectrometry-based approaches J Proteome Res 2009 8 4604 4614 10.1021/pr900420b 19673542

[B8] Khalil AA Biomarker discovery: a proteomic approach for brain cancer profiling Cancer Sci 2007 98 201 213 10.1111/j.1349-7006.2007.00374.x 17233837 PMC11158801

[B9] Saikali S Avril T Collet B Hamlat A Bansard JY Drenou B Expression of nine tumour antigens in a series of human glioblastoma multiforme: interest of EGFRvIII, IL-13Ralpha2, gp100 and TRP-2 for immunotherapy J Neurooncol 2007 81 139 148 10.1007/s11060-006-9220-3 17004103

[B10] Niclou SP Fack F Rajcevic U Glioma proteomics: Status and perspectives J Proteomics 2010 73 1823 1838 10.1016/j.jprot.2010.03.007 20332038

[B11] Gharbi S Gaffney P Yang A Zvelebil MJ Cramer R Waterfield MD Evaluation of two-dimensional differential gel electrophoresis for proteomic expression analysis of a model breast cancer cell system Mol Cell Proteomics 2002 1 91 98 10.1074/mcp.T100007-MCP200 12096126

[B12] Zhou G Li H DeCamp D Chen S Shu H Gong Y 2D differential in-gel electrophoresis for the identification of esophageal scans cell cancer-specific protein markers Mol Cell Proteomics 2002 1 117 124 10.1074/mcp.M100015-MCP200 12096129

[B13] Deighton RF McGregor R Kemp J McCulloch J Whittle IR Glioma pathophysiology: insights emerging from proteomics Brain Pathol 2010 20 691 703 10.1111/j.1750-3639.2010.00376.x 20175778 PMC8094634

[B14] Hiratsuka M Inoue T Toda T Kimura N Shirayoshi Y Kamitani H Proteomics-based identification of differentially expressed genes in human gliomas: down-regulation of SIRT2 gene Biochem Biophys Res Commun 2003 309 558 566 10.1016/j.bbrc.2003.08.029 12963026

[B15] Fleming CE Nunes AF Sousa MM Transthyretin: more than meets the eye Prog Neurobiol 2009 89 266 276 10.1016/j.pneurobio.2009.07.007 19665514

[B16] Park CK Jung JH Park SH Jung HW Cho BK Multifarious proteomic signatures and regional heterogeneity in glioblastomas J Neurooncol 2009 94 31 39 10.1007/s11060-009-9805-8 19219580

[B17] Leandro-Garcia LJ Leskela S Landa I Montero-Conde C Lopez-Jimenez E Leton R Tumoral and tissue-specific expression of the major human beta-tubulin isotypes Cytoskeleton (Hoboken) 2010 67 214 223 20191564 10.1002/cm.20436

[B18] Jung CS Foerch C Schanzer A Heck A Plate KH Seifert V Serum GFAP is a diagnostic marker for glioblastoma multiforme Brain 2007 130 3336 3341 10.1093/brain/awm263 17998256

[B19] Chumbalkar VC Subhashini C Dhople VM Sundaram CS Jagannadham MV Kumar KN Differential protein expression in human gliomas and molecular insights Proteomics 2005 5 1167 1177 10.1002/pmic.200401202 15759318

[B20] Ordys BB Launay S Deighton RF McCulloch J Whittle IR The role of mitochondria in glioma pathophysiology Mol Neurobiol 2010 42 64 75 10.1007/s12035-010-8133-5 20414816

[B21] Park CK Jung JH Moon MJ Kim YY Kim JH Park SH Tissue expression of manganese superoxide dismutase is a candidate prognostic marker for glioblastoma Oncology 2009 77 178 181 10.1159/000231888 19641337

[B22] Ria F Landriscina M Remiddi F Rosselli R Iacoangeli M Scerrati M The level of manganese superoxide dismutase content is an independent prognostic factor for glioblastoma. Biological mechanisms and clinical implications Br J Cancer 2001 84 529 534 10.1054/bjoc.2000.1594 11207049 PMC2363764

[B23] Corti S Locatelli F Papadimitriou D Donadoni C Salani S Del BR Identification of a primitive brain-derived neural stem cell population based on aldehyde dehydrogenase activity Stem Cells 2006 24 975 985 10.1634/stemcells.2005-0217 16293577

[B24] Deng S Yang X Lassus H Liang S Kaur S Ye Q Distinct expression levels and patterns of stem cell marker, aldehyde dehydrogenase isoform 1 (ALDH1), in human epithelial cancers PLoS One 2010 5 e10277 10.1371/journal.pone.0010277 20422001 PMC2858084

[B25] Ginestier C Hur MH Charafe-Jauffret E Monville F Dutcher J Brown M ALDH1 is a marker of normal and malignant human mammary stem cells and a predictor of poor clinical outcome Cell Stem Cell 2007 1 555 567 10.1016/j.stem.2007.08.014 18371393 PMC2423808

[B26] Rasheed ZA Yang J Wang Q Kowalski J Freed I Murter C Prognostic significance of tumorigenic cells with mesenchymal features in pancreatic adenocarcinoma J Natl Cancer Inst 2010 102 340 351 10.1093/jnci/djp535 20164446 PMC2831049

[B27] Charafe-Jauffret E Ginestier C Iovino F Tarpin C Diebel M Esterni B Aldehyde dehydrogenase 1-positive cancer stem cells mediate metastasis and poor clinical outcome in inflammatory breast cancer Clin Cancer Res 2010 16 45 55 10.1158/1078-0432.CCR-09-1630 20028757 PMC2874875

[B28] Son MJ Woolard K Nam DH Lee J Fine HA SSEA-1 is an enrichment marker for tumor-initiating cells in human glioblastoma Cell Stem Cell 2009 4 440 452 10.1016/j.stem.2009.03.003 19427293 PMC7227614

[B29] Tchoghandjian A Baeza N Colin C Cayre M Metellus P Beclin C A2B5 cells from human glioblastoma have cancer stem cell properties Brain Pathol 2010 20 211 221 10.1111/j.1750-3639.2009.00269.x 19243384 PMC8094826

[B30] Takano S Wadhwa R Yoshii Y Nose T Kaul SC Mitsui Y Elevated levels of mortalin expression in human brain tumors Exp Cell Res 1997 237 38 45 10.1006/excr.1997.3754 9417864

[B31] Wadhwa R Takano S Kaur K Deocaris CC Pereira-Smith OM Reddel RR Upregulation of mortalin/mthsp70/Grp75 contributes to human carcinogenesis Int J Cancer 2006 118 2973 2980 10.1002/ijc.21773 16425258

[B32] Iwadate Y Sakaida T Hiwasa T Nagai Y Ishikura H Takiguchi M Molecular classification and survival prediction in human gliomas based on proteome analysis Cancer Res 2004 64 2496 2501 10.1158/0008-5472.CAN-03-1254 15059904

[B33] Zhang R Tremblay TL McDermid A Thibault P Stanimirovic D Identification of differentially expressed proteins in human glioblastoma cell lines and tumors Glia 2003 42 194 208 10.1002/glia.10222 12655603

[B34] Garrido C Brunet M Didelot C Zermati Y Schmitt E Kroemer G Heat shock proteins 27 and 70: anti-apoptotic proteins with tumorigenic properties Cell Cycle 2006 5 2592 2601 10.4161/cc.5.22.3448 17106261

[B35] Odreman F Vindigni M Gonzales ML Niccolini B Candiano G Zanotti B Proteomic studies on low- and high-grade human brain astrocytomas J Proteome Res 2005 4 698 708 10.1021/pr0498180 15952716

[B36] Coe H Michalak M ERp57, a multifunctional endoplasmic reticulum resident oxidoreductase Int J Biochem Cell Biol 2010 42 796 799 10.1016/j.biocel.2010.01.009 20079872

[B37] Fountoulakis M Hardmaier R Schuller E Lubec G Differences in protein level between neonatal and adult brain Electrophoresis 2000 21 673 678 10.1002/(SICI)1522-2683(20000201)21:3<673::AID-ELPS673>3.0.CO;2-Y 10726776

[B38] Lubec G Nonaka M Krapfenbauer K Gratzer M Cairns N Fountoulakis M Expression of the dihydropyrimidinase related protein 2 (DRP-2) in Down syndrome and Alzheimer's disease brain is downregulated at the mRNA and dysregulated at the protein level J Neural Transm Suppl 1999 57 161 177 10666674 10.1007/978-3-7091-6380-1_10

[B39] Weitzdoerfer R Fountoulakis M Lubec G Aberrant expression of dihydropyrimidinase related proteins-2,-3 and -4 in fetal Down syndrome brain J Neural Transm Suppl 2001 95 107 11771764 10.1007/978-3-7091-6262-0_8

[B40] Yoshida H Watanabe A Ihara Y Collapsin response mediator protein-2 is associated with neurofibrillary tangles in Alzheimer's disease J Biol Chem 1998 273 9761 9768 10.1074/jbc.273.16.9761 9545313

[B41] Byk T Ozon S Sobel A The Ulip family phosphoproteins--common and specific properties Eur J Biochem 1998 254 14 24 10.1046/j.1432-1327.1998.2540014.x 9652388

[B42] Choi YL Kim CJ Matsuo T Gaetano C Falconi R Suh YL HUlip, a human homologue of unc-33-like phosphoprotein of Caenorhabditis elegans; Immunohistochemical localization in the developing human brain and patterns of expression in nervous system tumors J Neurooncol 2005 73 19 27 10.1007/s11060-004-3013-3 15933812

[B43] Gu Y Hamajima N Ihara Y Neurofibrillary tangle-associated collapsin response mediator protein-2 (CRMP-2) is highly phosphorylated on Thr-509, Ser-518, and Ser-522 Biochemistry (Mosc) 2000 39 4267 4275 10.1021/bi992323h 10757975

[B44] Cole AR Causeret F Yadirgi G Hastie CJ McLauchlan H McManus EJ Distinct priming kinases contribute to differential regulation of collapsin response mediator proteins by glycogen synthase kinase-3 in vivo J Biol Chem 2006 281 16591 16598 10.1074/jbc.M513344200 16611631 PMC1805471

[B45] Kobeissy FH Ottens AK Zhang Z Liu MC Denslow ND Dave JR Novel differential neuroproteomics analysis of traumatic brain injury in rats Mol Cell Proteomics 2006 5 1887 1898 10.1074/mcp.M600157-MCP200 16801361

[B46] Setsuie R Wada K The functions of UCH-L1 and its relation to neurodegenerative diseases Neurochem Int 2007 51 105 111 10.1016/j.neuint.2007.05.007 17586089

[B47] Karp NA Lilley KS Maximising sensitivity for detecting changes in protein expression: experimental design using minimal CyDyes Proteomics 2005 5 3105 3115 10.1002/pmic.200500083 16035117

[B48] Engelen K Sifrim A Van de PB Laukens K Arckens L Marchal K Alternative experimental design with an applied normalization scheme can improve statistical power in 2D-DIGE experiments J Proteome Res 2010 9 4919 4926 10.1021/pr100010u 20681517

[B49] McDonald JH Handbook of Biological Statistics 2009 2 Sparky House Publishing, Baltimore, Maryland. Ref Type: Generic 118 122

[B50] Rolland AD Evrard B Guitton N Lavigne R Calvel P Couvet M Two-dimensional fluorescence difference gel electrophoresis analysis of spermatogenesis in the rat J Proteome Res 2007 6 683 697 10.1021/pr060436z 17269725

[B51] Romero-Calvo I Ocon B Martinez-Moya P Suarez MD Zarzuelo A Martinez-Augustin O Reversible Ponceau staining as a loading control alternative to actin in Western blots Anal Biochem 2010 401 318 320 10.1016/j.ab.2010.02.036 20206115

